# Altered periodic and aperiodic activities in patients with disorders of consciousness

**DOI:** 10.3389/fnins.2025.1657792

**Published:** 2025-10-22

**Authors:** Lihui Cai, Yujie Li, Zhelun Cheng, Yueqing Dong

**Affiliations:** ^1^School of Life Sciences, Tiangong University, Tianjin, China; ^2^Jinnan Hospital, Tianjin University, Tianjin, China

**Keywords:** disorders of consciousness, electroencephalogram, power spectrum, aperiodic activity, variability

## Abstract

**Introduction:**

Disorders of consciousness (DoC), including unresponsive wakefulness syndrome (UWS) and minimally conscious state (MCS), are primarily diagnosed behaviorally. Recent evidence indicates that loss of consciousness manifests as irregularities in neural oscillatory activity across delta, theta, and alpha frequency bands. However, conventional spectral analysis often conflates periodic oscillations with aperiodic 1/*f* components, potentially obscuring consciousness-related dynamics.

**Methods:**

To elucidate the mechanistic basis of spectral alterations in consciousness impairment, we compared oscillatory and aperiodic activity patterns in the electroencephalogram (EEG) of patients with different consciousness levels. We further examined the spatiotemporal variability of these neural signatures and rigorously evaluated their discriminative power for state classification using support vector machine (SVM) analysis.

**Results:**

While periodic and aperiodic activities are independent, our results indicate that both components exhibit significant differences between groups at both local and global scales. Critically, higher spatial and temporal variability of aperiodic features (spectral exponent) were correlated with preserved consciousness. When distinguishing UWS from MCS, the combination of periodic and aperiodic features significantly improved classification performance compared to using either metric alone.

**Discussion:**

Our findings demonstrate that both periodic oscillations and aperiodic activity provide valuable information about consciousness levels. Critically, the spatiotemporal dynamics of the aperiodic component serve as a key marker of brain state. This underscores the necessity of accounting for aperiodic activity in mechanistic studies and clinical assessments of DoC.

## 1 Introduction

Disorders of consciousness (DoC) are often the outcomes of severe brain injuries, manifesting as a wide-ranging spectrum of conditions. Among them, the vegetative state (VS), also referred to as unresponsive wakefulness syndrome (UWS) (Laureys et al., 2010; Jennett and Plum, 1972), represents a state where patients are completely devoid of awareness. In contrast, patients in the minimally conscious state (MCS) exhibit intermittent but distinct signs of external awareness (Giacino et al., 2002). In clinical practice, accurately distinguishing between UWS and MCS is of utmost significance as it serves as the foundation for formulating suitable treatment strategies. The differential diagnosis between these states is predominantly grounded in behavioral assessments. Currently, the Coma Recovery Scale—Revised (CRS-R) stands as the gold-standard tool for this purpose (Kalmar and Giacino, 2007). However, recent developments suggest that relying solely on behavioral evaluation might not be sufficient. Brain-imaging technologies have revealed that around 15–25% of patients who seem unresponsive clinically exhibit residual signs of awareness (Cruse et al., 2011; King et al., 2013; Owen et al., 2006). Among the various neuroimaging tools, electroencephalogram (EEG) is a widely applicable, less expensive, and suitable procedure that provides direct and immediate information about the consciousness states.

Growing evidence highlights the distinct roles of EEG signatures in DoC, as demonstrated by studies spanning spectral analysis (Schiff et al., 2014; Lutkenhoff et al., 2020; Wutzl et al., 2021). The most robust findings center on spectral power alterations across canonical frequency bands: delta (1–4 Hz), theta (4–8 Hz), alpha (8–12 Hz), and beta (15–30 Hz). DoC patients exhibit elevated delta and theta power alongside suppressed alpha power compared to healthy controls, with this alpha suppression serving as a characteristic marker of impaired consciousness (Lechinger et al., 2013; Sitt et al., 2014; Naro et al., 2016). Notably, patients in MCS show significantly higher alpha power than those in UWS, particularly in the central, parietal, and occipital regions (Bai et al., 2021b). Furthermore, the changes in consciousness levels may be linked to the shift of dominant spectral peaks within the electroencephalogram (EEG), as illustrated by the “ABCD” model (Schiff, 2016; Edlow et al., 2020). This model organizes EEG power spectra into four broad categories. The EEG patterns corresponding to these different categories offer crucial insights into the degree of thalamocortical deafferentation and aid in the diagnosis of consciousness levels. Notably, the behavioral diagnosis of unresponsive wakefulness syndrome (UWS) or minimally conscious state (MCS) can be associated with more than one spectral category. For example, a patient with UWS may display either A-type or B-type dynamics. On the other hand, not all EEG spectrals fit with ABCD categories. These factors impede the application of this model in the clinical field.

The vast majority of the narrowband analysis presumes that spectral power implies oscillatory power and overlook the existence of aperiodic activity. In fact, the neural power spectrum encompasses not only oscillatory activity but also the aperiodic 1/*f* component. Recent methodological advancements have revealed that band-limited variations in oscillatory power can be swayed by alterations in the aperiodic exponent (Donoghue et al., 2020, 2021). Put simply, the detected changes in neural power might not mirror actual changes in the periodic signal; instead, they could be due to changes in aperiodic features, or a combination of both. Furthermore, aperiodic activity, which has frequently been regarded as either noise or a bothersome variable, actually has significant demographic and clinical correlations, as well as physiological implications (Voytek et al., 2015; Roche et al., 2019; Salvatore et al., 2024; Pani et al., 2022). For example, aperiodic analysis can detect baseline neurophysiological changes that reflect overall alterations in cortical excitability, synaptic activity, and metabolic demands (Deodato and Melcher, 2024). These factors are closely associated with the maintenance or transformation of consciousness states. Recent research has demonstrated that the characteristics of aperiodic electroencephalogram (EEG) carry information related to consciousness, such as the depth of anesthesia and sleep stage (Favaro et al., 2023; Widmann et al., 2025; Kreuzer et al., 2020). Nevertheless, the diagnostic potential of aperiodic features in evaluating the consciousness levels of DoC patients remains largely unexplored.

To address this gap, the present study aims to systematically evaluate the diagnostic value of both periodic and aperiodic EEG components in distinguishing between patients with UWS and MCS. We hypothesize that both components are associated with consciousness states. To test this, we decompose the original neural spectrum of scalp EEG into periodic and aperiodic components and obtain corresponding features. We then explore the differences in these components between UWS and MCS patients on both global and regional scales, with particular attention to their spatial and temporal variability. Finally, we examine the utility of both components in distinguishing between patients with different consciousness levels using multiple machine learning algorithms. Ultimately, this work seeks to establish a novel biomarker framework derived from the neural power spectrum, providing a more mechanistic basis for the diagnosis and stratification of DoC.

## 2 Materials and methods

### 2.1 Participants

Electroencephalogram (EEG) data were collected from 47 patients with DoC (22 males; mean age 42 ± 15.13 years) recruited from Department of Neurosurgery at Tianjin Kanghui Hospital, including etiological subtypes of 18 anoxic brain injury cases, 12 traumatic brain injuries, and 12 cerebrovascular accidents. The study protocol received approval from the institutional Ethics Committee, with written informed consent obtained from all legal guardians. Exclusion criteria comprised disease duration <1 month, scalp lesions, or intracranial metallic implants. Concurrent with EEG recordings, a certified neurologist systematically evaluated consciousness levels using the Coma Recovery Scale-Revised (CRS-R). To ensure diagnostic accuracy, each patient underwent a minimum of four behavioral assessments, with final diagnoses determined by optimal behavioral responses. The temporal proximity between EEG recordings and behavioral assessments was maintained within a one-week window. Based on CRS-R performance, participants were stratified into two clinical subgroups: UWS (*n* = 27) and MCS (*n* = 20). Statistical analyses revealed no significant inter-group differences in age, gender distribution, etiology profiles, or disease chronicity. Comprehensive demographic and clinical characteristics are summarized in Table 1. Written informed consent was obtained from legally authorized representatives of DoC patients.

**Table 1:** Comparison of socio-demographic and clinical data between patients with UWS and MCS.

Subject characteristics	UWS	MCS	*p*-value
Age (years) (mean ± SD)	46.22 ± 13.58	48.9 ± 12.04	0.42
Gender (number)	Male: 19	Male: 14	1
	Female: 8	Female: 6	
Etiology (number)	A: 5	A: 2	0.102
	H: 7	H: 8	
	T: 12	T: 6	
	O: 3	O: 4	
Time since injury (month) (mean ± SD)	10.67 ± 8.11	6.55 ± 4.29	0.23

A, s anoxia; H, hemorrhage; T, trauma; O, other causes; SD, standard deviation.

### 2.2 Data recording and preprocessing

All participants underwent resting—state EEG recordings that lasted for a minimum of 15 min. These recordings were carried out using a Neurosoft EEG system, which adopted a 30—channel electrode configuration following the international 10–20 system. The electrode sites included FP1, FP2, F3, F4, F7, F8, C3, C4, T7, T8, P3, P4, P7, P8, O1, O2, FZ, CZ, PZ, OZ, FT7, FT8, FC3, FC4, CP3, CP4, TP7, TP8, FCZ, and CPZ. Additionally, bilateral mastoid reference electrodes (A1/A2) were utilized. Before data acquisition, automated impedance checks ensured all channels remained below 5 kΩ, with signals sampled at 500 Hz. The raw EEG data underwent sequential preprocessing to optimize signal integrity. Machine learning-based Artifact Subspace Reconstruction (ASR) was then applied to dynamically identify and correct transient high-amplitude artifacts (e.g., electrode pops or movement-induced bursts) by statistically modeling clean EEG subspaces and reconstructing corrupted segments. Subsequently, Independent Component Analysis (ICA) employing the Infomax algorithm decomposed the multichannel signals into spatially independent components. Artifact-related components were automatically classified via the ICLABEL toolbox, which leverages a pretrained neural network to assign probabilistic labels. The cleaned data were reconstructed and re-referenced to the average reference.

Following automated processing, a certified neurophysiology technician performed visual inspection to manually discard epochs with residual non-physiological artifacts. For each participate, over 5 min of artifact-free data were retained. The validated signals were partitioned into consecutive 10-s non-overlapping epochs for downstream analysis. The entire preprocessing pipeline was executed using the EEGLAB toolbox (v2022.1).

### 2.3 Data analysis

The multitaper method (MTM) was employed to estimate the power spectral density (PSD) of the preprocessed EEG across electrodes and epochs, following Thomson’s orthogonal taper approach (Thomson, 1982). Compared to other approaches like Welch’s method, MTM offers three key advantages: it achieves a superior balance between frequency resolution and variance reduction via multiple orthogonal tapers, avoiding the frequency resolution loss from Welch’s signal segmentation; it is more robust to non-stationary EEG fluctuations (e.g., irregular slow waves in patients) by minimizing segment-boundary artifacts and leakage, unlike Welch’s rigid segmentation; and it enhances SNR for low-amplitude neural signals, critical for detecting subtle group differences. The MTM was implemented using the *pmtm* function in MATLAB (R2022b). Analysis was performed on non-overlapping 5-s windows, a duration selected to ensure stationarity while maintaining sufficient frequency resolution for capturing neural oscillatory dynamics. We employed a time-half bandwidth product of NW = 4, yielding 7 orthogonal Slepian tapers. This configuration provides an optimal balance between spectral leakage suppression and variance control, in accordance with established practices in multitaper spectral analysis (Babadi and Brown 2014; Prerau et al., 2017). No detrending was applied prior to spectral estimation. The NFFT size was set to the default value of 4,096. Finally, the individual tapered estimates were combined using Thomson’s adaptive weighting method to optimize the bias-variance tradeoff, which is the default procedure in the *pmtm* function when a single output is requested. All spectral estimates were conducted within a frequency range of 0.1 to 30 Hz, considering that the gamma band has been rarely considered in DOC studies and no discrimination between UWS and MCS was reported (Bai et al., 2021a,b).

The FOOOF Python package was then utilized to parametrize the neural power spectra by decomposing them into periodic oscillatory components and aperiodic 1/*f*-like dynamics. The aperiodic component was modeled as an exponential function: $L\left(f\right)=b-\log\left(k+F^{\chi }\right)$ , where b represents the broadband “offset,” k denotes the “knee” and the “exponent” χ quantifying the slope of the 1/*f* decay. Within the 0.1–30 Hz range, the algorithm was fitted using the fixed aperiodic mode with peak width limits of [0.5, 12], max_n_peaks = 3, min_peak_height = 0.01, and peak_threshold = 0.01. The goodness-of-fit of the final model was assessed by computing frequency-wise differences between the raw spectra and the final model fits, as well as by calculating the *R*-squared value. For further analysis, all models achieved *R*^2^ values exceeding 0.95, which is in accordance with previous studies (Wang et al., 2022, 2024). Both the aperiodic offset and exponent were extracted for each participant and subsequently used in statistical analyses.

Following the removal of the aperiodic component, the three dominant peak parameters (periodic metrics) of the neural spectrum were obtained, including the central frequency (CF), power over the aperiodic component (PW), and bandwidth (BW) of the peak. These parameters were also derived using multitaper-based PSD for further comparison. All periodic and aperiodic metrics were computed for each epoch and electrode independently. For each participant, the metrics were averaged to evaluate the oscillatory and aperiodic dynamics at both global (whole brain) and local (regional) spatial scales. Additionally, the temporal and spatial variability of these metrics was investigated by calculating the coefficients of variation (CV), defined as the ratio of the standard deviation to the mean value of the metrics across epochs or electrodes.

### 2.4 Statistical analysis

First, group differences in demographic characteristics (age, gender, etiology, and disease duration) were examined. Categorical variables (gender and etiology) were analyzed using chi-square tests, while continuous variables (age and disease duration) were assessed via one-way analysis of variance (ANOVA). For group comparisons of EEG metrics, Wilcoxon signed-rank tests were applied to channel-averaged data. To address channel-wise comparisons across 30 electrodes, *p*-values were adjusted for multiple comparisons using the false discovery rate (FDR) correction. Additionally, Spearman’s rank correlation analysis was performed to evaluate associations between metrics showing significant group differences and CRS-R scores. All statistical analyses were conducted in SPSS 25.0 (SPSS Inc., Chicago, IL, USA), with a significance threshold of *α* = 0.05.

### 2.5 Classification

To further evaluate the ability of periodic or aperiodic features in distinguishing between UWS and MCS patients, we conducted the classification analysis using a support vector machine approach. To reduce the workload of classification, only the global-scale features (features averaged across all channels) with significant group differences (*p* < 0.05) were selected as the input of the classifiers. A double repeated stratified cross-validation framework was employed to ensure robust evaluation reliability of the classification model. First, we implemented 10 repetitions of 5-fold stratified cross-validation during the hyperparameter tuning phase with a grid search strategy. The dataset is partitioned into 5 mutually exclusive subsets of equal or comparable size, referred to as folds. In each experimental cycle, the model is trained on 4 folds and validated on the remaining fold. This process iterates 5 times, with the final performance metric calculated as the mean across all 5 iterations. Each “fold” maintains the sample ratio of each category as closely as possible to the class proportion in the original dataset. After identifying the optimal parameter configuration, an independent validation phase was conducted using 50 repetitions of 5-fold cross-validation to reduce stochastic variability induced by random data partition. Finally, the averaged accuracy, sensitivity, specificity and Area Under the Curve (AUC) were calculated to assess the classification performance.

## 3 Results

**Figure 1:**
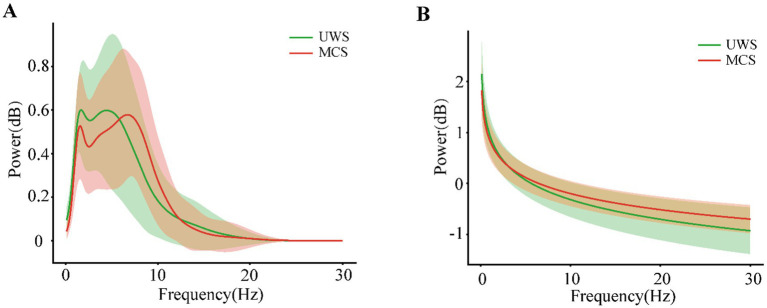
The periodic **(A)** and aperiodic **(B)** components of the power spectral density averaged across the individuals with UWS or MCS. The shaded areas indicate the standard deviation of power across the frequencies.

Figures 1A,B illustrated the grand-averaged full-scalp power spectra with only periodic or aperiodic component reserved, respectively for UWS and MCS group, respectively. Compared to the patients with UWS, the MCS patients exhibited lower delta and higher alpha oscillation power with the second peak shifting right. While for aperiodic activity, the MCS patients showed higher power especially within the range from 5 to 30 Hz.

**Figure 2:**
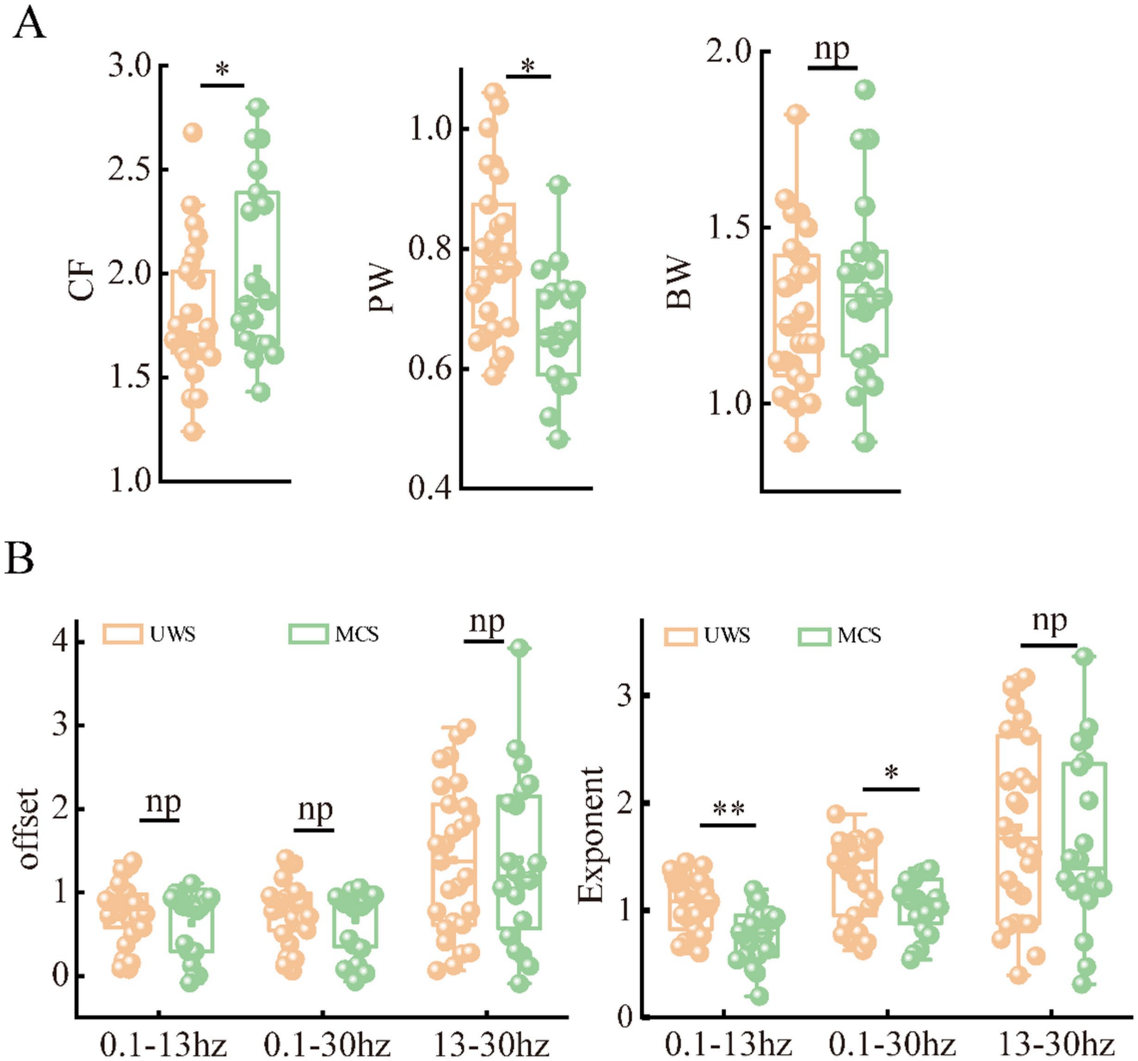
Differences of periodic **(A)** and aperiodic **(B)** metrics on global scale (averaged across the channels) between patients with UWS and MCS. * denotes *p* < 0.05, ** denotes *p* < 0.005 and *np* denotes *p* > 0.05.

To better characterize the alterations of periodic and aperiodic activities with consciousness levels, we compared the corresponding parameters for both components between the two groups on a global scale, shown in Figure 2 and Table 2. The MCS group (Mean: 2.10, SD:0.55) exhibited significant higher center frequency (*p* = 0.042) than the UWS group (Mean: 1.79, SD:0.32). On the contrary, the UWS patients (Mean: 0.79, SD:0.13) have higher peak power (*p* = 0.027) than those with MCS (Mean: 0.72, SD:0.17). No significant difference was observed for the bandwidth between groups (*p* = 0.287). In the raw power spectra, the MCS group showed increased relative power in theta (*p* = 0.0027), alpha (*p* = 0.0004) and beta (*p* = 0.029) bands compared with UWS, while an opposite trend was found in delta band (*p* = 0.0004). While in the periodic spectrum, only the delta and alpha bands (*p* = 0.034) showed significant group differences. Considering that the loss of consciousness may have different effects on the spectral slope (exponent) in low and high frequency bands, we compared the aperiodic metrics within different frequency ranges. In the broad frequency range of 0.1–30 Hz, the exponents were significantly reduced (*p* = 0.015) in the MCS patients (Mean: 1.05, SD: 0.27) compared to those with UWS (Mean: 1.29, SD: 0.37). A similar but more obvious change of exponents can be found in the 0.1–13 Hz range. While in the 13–30 Hz range, no significant difference was found. For the offsets, results showed no group differences in all the frequency range. Thus, further analysis was mainly performed within the 0.1–13 Hz range.

**Table 2:** Comparison of channel-averaged spectral features.

Features	UWS	MCS	*U* value	*p*-value
CF	1.79 ± 0.317	2.104 ± 0.55	175	0.042
PW	0.79 ± 0.131	0.716 ± 0.171	373	0.027
BW	1.254 ± 0.222	1.334 ± 0.258	220	0.287
Delta raw power	0.719 ± 0.091	0.572 ± 0.135	443	0.0002
Theta raw power	0.163 ± 0.054	0.226 ± 0.066	126	0.002
Alpha raw power	0.049 ± 0.026	0.099 ± 0.049	89	0.0001
Beta raw power	0.069 ± 0.069	0.103 ± 0.083	168	0.029
Delta periodic power	0.293 ± 0.117	0.208 ± 0.084	387	0.012
Theta periodic power	0.363 ± 0.198	0.397 ± 0.163	241	0.540
Alpha periodic power	0.123 ± 0.084	0.199 ± 0.099	159	0.017
Beta periodic power	0.221 ± 0.173	0.196 ± 0.114	268	0.974
0.1–13 Hz offset	0.763 ± 0.363	0.635 ± 0.392	300	0.53
0.1–30 Hz offset	0.793 ± 0.373	0.671 ± 0.384	294	0.61
13–30 Hz offset	1.387 ± 0.888	1.39 ± 1.027	277	0.89
0.1–13 Hz exponent	1.06 ± 0.255	0.767 ± 0.26	424	0.00096
0.1–30 Hz exponent	1.289 ± 0.365	1.05 ± 0.265	383	0.015
13–30 Hz exponent	1.769 ± 0.855	1.63 ± 0.808	294	0.61

**Figure 3:**
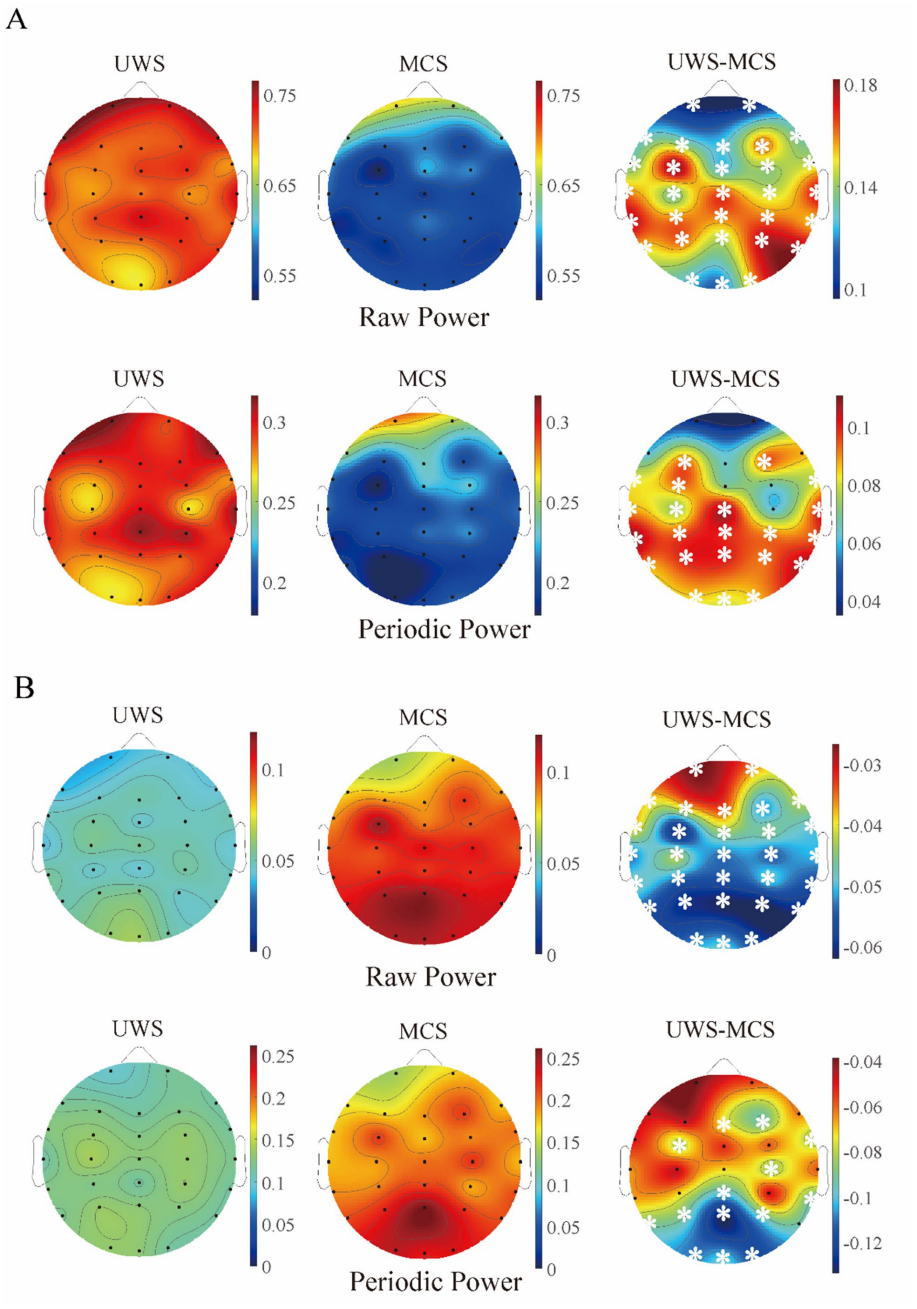
The scalp topography of raw and periodic (relative) power in different groups. **(A)** Delta band; **(B)** alpha band. The third column exhibits the difference between UWS and MCS group, with the white stars marked the channels with significant group differences (*p* < 0.05, FDR corrected).

**Figure 4:**
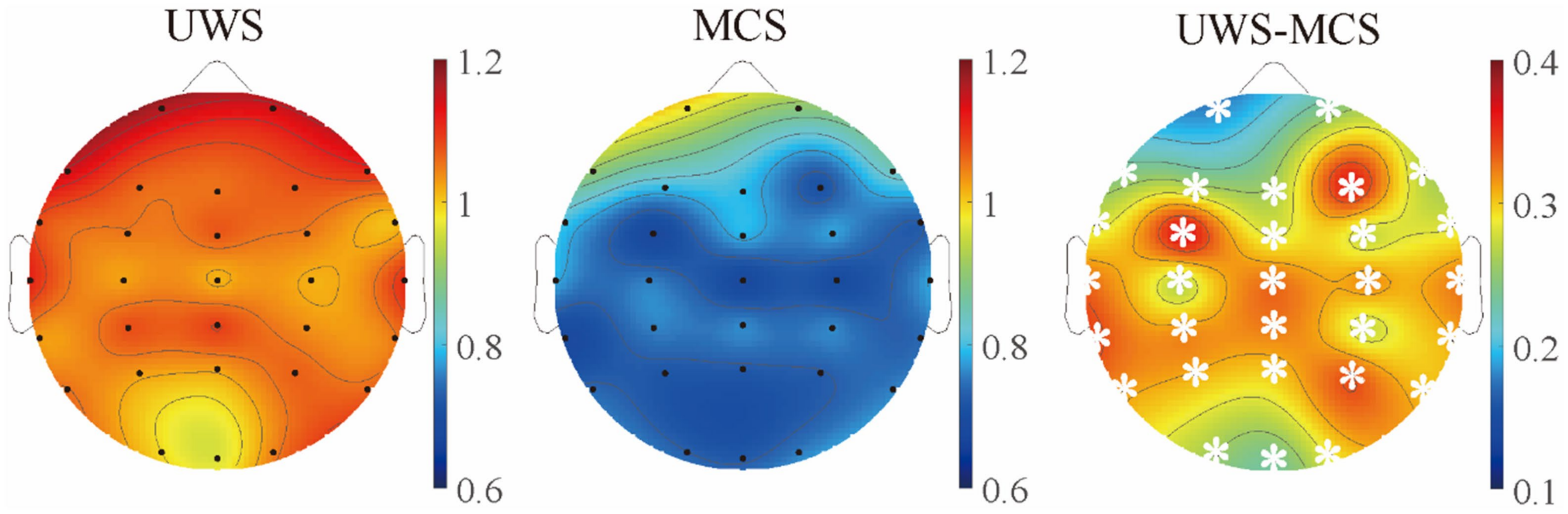
The scalp topography of spectral exponents in different groups. The third column exhibits the difference between UWS and MCS group, with the white stars marked the channels with significant group differences (*p* < 0.05, FDR corrected).

In addition, we conducted pairwise group comparisons on a local scale for different channels or brain areas. Though the MCS group showed higher mean center frequency and lower mean peak power in most channels than UWS, no significant difference was found in any channel after post-correction. Similar to the spatial distribution of raw spectrum, the human brain was more likely to show higher delta periodic power in the frontal area and alpha periodic power in the occipital area for both UWS and MCS patients. Significant decrease of delta power could be found in the whole brain except the frontal area from UWS to MCS. The alpha power also exhibited significant differences in most channels especially in the occipital area (Figure 3). In the 0.1–13 Hz range, the UWS group displayed the highest exponents in the frontal area and the lowest values in the occipital area. Similar spatial distribution could be found for the MCS group. *Post hoc* comparisons showed that UWS and MCS differed significantly in exponent at the whole brain with the strongest difference found at the central area (Figure 4).

**Figure 5:**
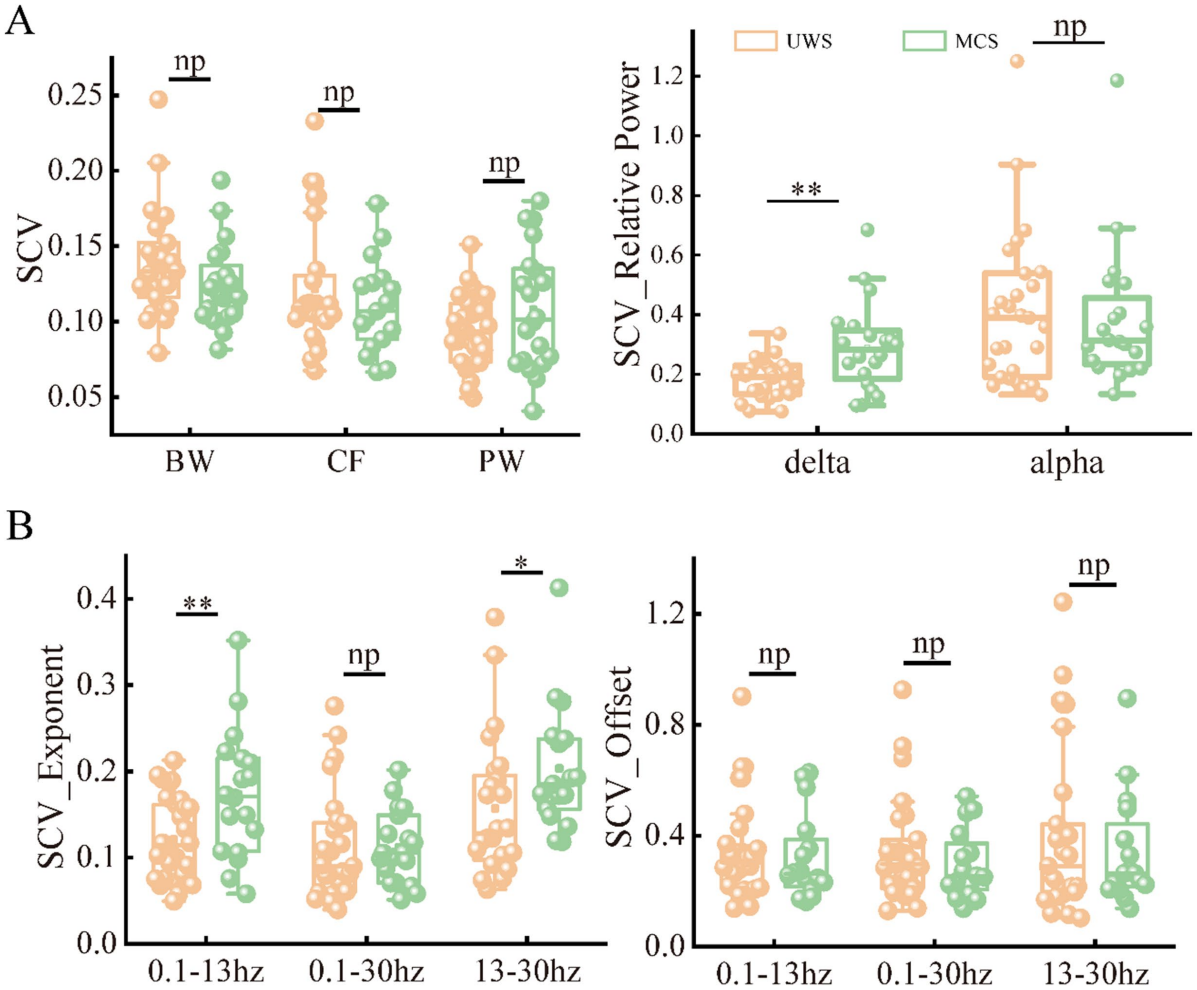
The spatial variability of periodic **(A)** and aperiodic **(B)** metrics in the patients with UWS and MCS. * denotes *p* < 0.05, ** denotes *p* < 0.005 and *np* denotes *p* > 0.05. SCV, spatial CV.

**Figure 6:**
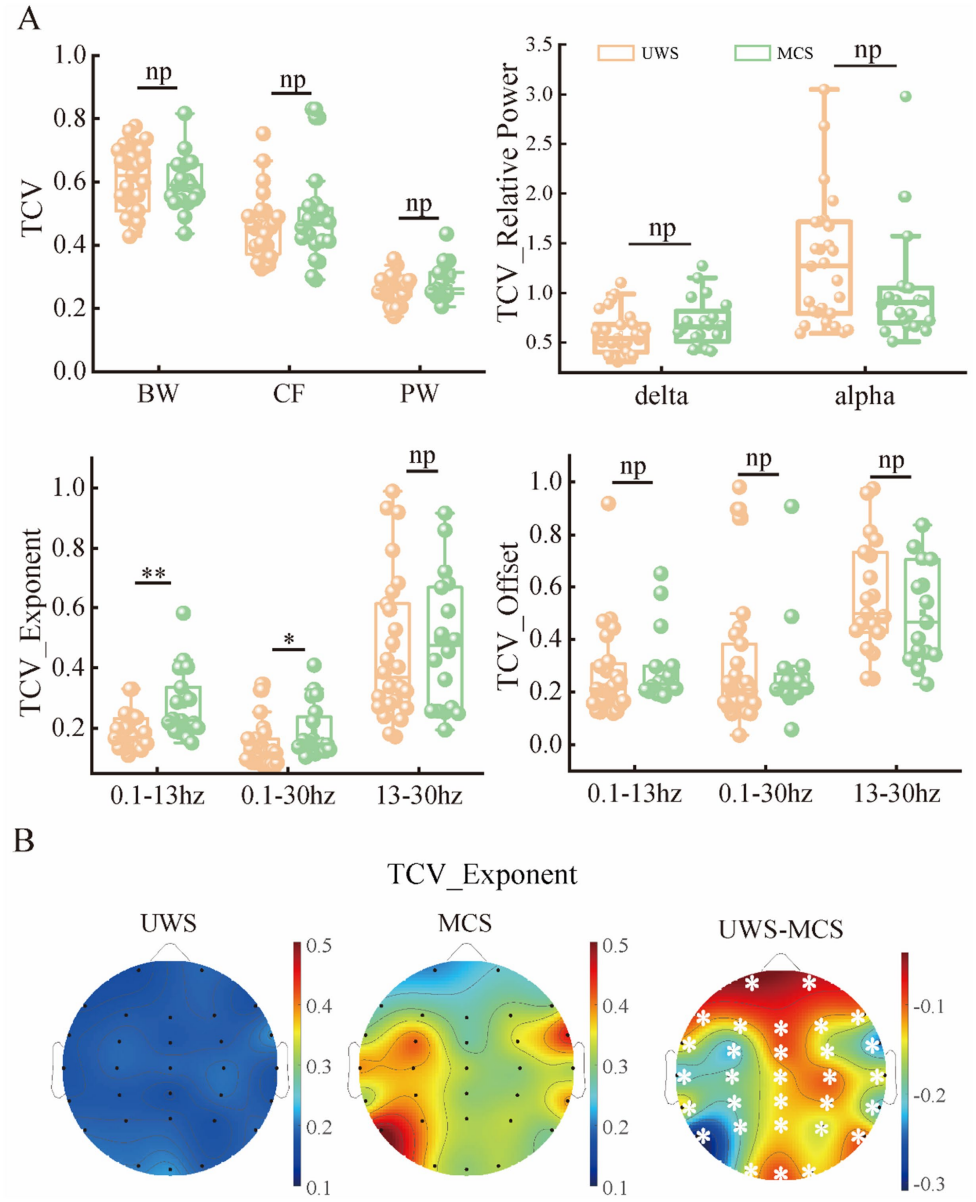
The temporal variability of periodic and aperiodic metrics in the patients with UWS and MCS. **(A)** The temporal variability of the metrics global scale. **(B)** The scalp topography of CVs for exponents in different groups. * denotes *p* < 0.05, ** denotes *p* < 0.005 and *np* denotes *p* > 0.05. TCV: spatial CV. All the channels showed significant group differences (*p* < 0.05, FDR corrected) of CVs for exponents.

As the metrics were uniformly distributed among brain regions, we further explored the spatial variability of periodic and aperiodic metrics using spatial CV, shown in Figure 5. There were no significant differences of CV values between UWS and MCS patients for parameters CF, PW and BW, whereas the delta power showed higher spatial CVs (*p* < 0.005) in MCS patients than those in UWS. The MCS group showed higher spatial variability of exponents than UWS in both 0.1–13 Hz and 13–30 Hz range, whereas the CVs of exponents in the 0.1–30 Hz range did not differ between groups. We further investigate the temporal fluctuation of the metrics on global and local scales. No significant group differences were found for the periodic parameters and offset. While increased temporal variability of exponents was observed in the 0.1–13 Hz and 0.1–30 Hz range. On local scale, both UWS and MCS group showed the highest CVs in the posterior area. Significant increase of temporal CVs can be found in the whole brain after post-correction (Figure 6).

**Figure 7:**
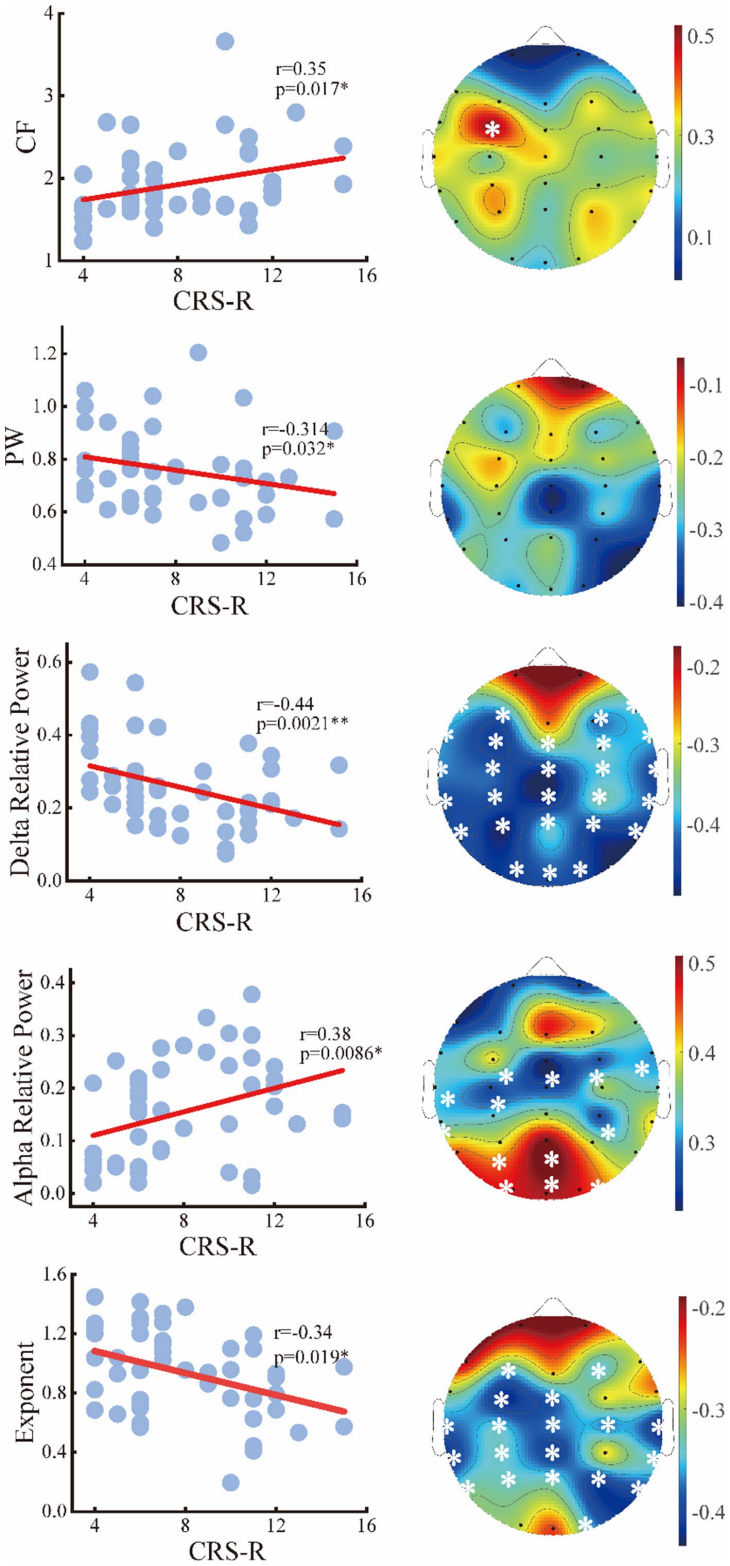
Relation between the periodic or aperiodic metrics and CRS-R score of the patients on global scale (left panel) and local scale (right panel). The white stars marked the channels with significant correlations.

**Figure 8:**
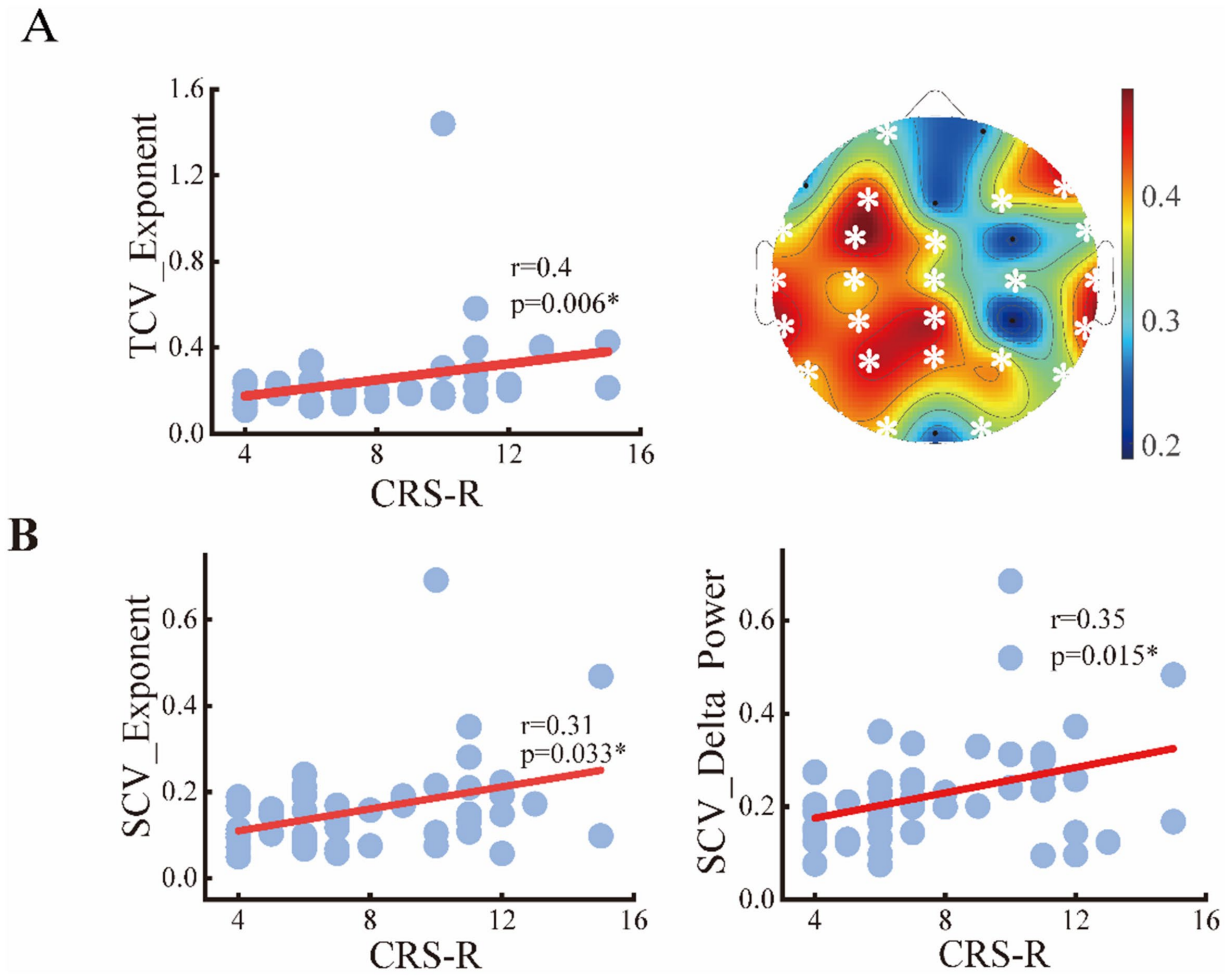
Relation between the spatial or temporal variability of metrics and CRS-R score of the patients. **(A)** Temporal CVs of exponents on global and local scale; **(B)** spatial CVs of exponents and delta power.

To assess the relationships between the periodic or aperiodic activities with the behavioral performance of DoC patients, we estimated the Spearman correlations between the abovementioned metrics and CRS-R scores on local and global scales, respectively. As expected, a positive correlation between center frequency and CRS-R score was found on global scale (*r* = 0.35, *p* = 0.034), with the C3 channel (left central area) only showed significant correlation on the local scale. While the peak power was negatively correlated with the CRS-R score (*r* = −0.314, *p* = 0.034) only on a global scale. The delta/alpha power showed significant correlations with CRS-R score especially in the posterior area, though an opposite trend can be observed. While for the aperiodic metrics, the exponent also had a negative correlation with the CRS-R score (*r* = −0.34, *p* = 0.019) (Figure 7). On the contrary, the temporal CVs were positively correlated with the behavioral performance (*r* = 0.4, *p* = 0.006), with the strongest correlation found in the central and parietal areas. The individuals with higher spatial CVs of exponent (*r* = 0.31, *p* = 0.033) and delta power (*r* = 0.35, *p* = 0.015) were also more likely to have better behavioral performance (Figure 8).

Finally, we assessed the diagnostic utility of global features in detecting consciousness levels using an SVM classifier. Among aperiodic metrics with significant intergroup differences, the temporal CVs of the spectral exponent within the 0.1–13 Hz frequency band demonstrated the strongest classification performance, yielding an accuracy of 70.6% and an AUC of 0.782. For periodic features, the spatial CVs of delta power achieved the optimal classification results, with an accuracy of 70.0% and an AUC of 0.762. However, both features demonstrate poor ability in detecting MCS, manifested by the low sensitivity value (44.5 and 47.7%) when the highest accuracy was obtained. Significantly, integrating both periodic and aperiodic features enhanced classification performance, achieving an accuracy of 78.2% and an AUC of 0.861. Moreover, the identification power of MCS was remarkably elevated with a sensitivity of 77.7% (see Table 3).

**Table 3:** Classification performance obtained with the periodic or aperiodic features on global scale.

Features	ACC	SEN	SPE	AUC
Delta relative power	65.0	51.7	74.7	0.697
Alpha relative power	66.9	60.3	71.8	0.701
CF	65.1	36.5	86.3	0.680
PW	62.9	43.2	77.3	0.702
Exponent (0.1–13 Hz)	67.5	56.2	76.0	0.760
TCV of exponent (0.1–13 Hz)	70.6	44.5	89.9	0.782
SCV of exponent (0.1–13 Hz)	68.1	48.7	82.4	0.754
SCV of exponent (13–30 Hz)	67.3	68.0	67.0	0.693
SCV of delta relative power	70.0	47.7	86.5	0.762
Combined features	78.2	77.7	78.3	0.861

## 4 Discussion

This study aimed to explore the roles of the aperiodic and periodic components of the scalp EEG in detecting consciousness levels. Our results revealed a significant difference in periodic activity (denoted by the center frequency, peak power of the periodic power spectra and alpha power) between the patients with UWS and MCS. Compared to the UWS group, the MCS group demonstrated significantly lower exponents especially on the whole brain. Moreover, enhanced spatial and temporal variability of the exponents could be observed from UWS to MCS. Combing periodic and aperiodic parameters could improve the diagnosis performance in distinguishing UWS and MCS compared to that with only periodic features. These results indicate the non-negligible role of aperiodic components of brain activity in assessing residual consciousness. Our results revealed significantly lower delta power but higher alpha power with the traditional approach in MCS group compared to the UWS group. This is consistent with the previous spectral studies that linked enhanced delta and suppressed alpha activities with low consciousness level (Sitt et al., 2014; Stefan et al., 2018; Lehembre et al., 2012). In addition, this study showed increased power in the theta band in UWS patients compared with MCS, which is in accordance with the previous studies that reported positive correlations between power and consciousness state (Piarulli et al., 2016). However, after removing the aperiodic component, only the oscillatory power in delta and alpha band showed significant group differences. This suggests that the apparent changes in narrowband power could be attributed to both the reductions in true oscillatory power and changes in aperiodic exponent. The strong correlation between loss of consciousness and elevated delta power is attributed to widespread cortical deactivation during the ‘down states’ of slow oscillations (Frohlich et al., 2021). Supporting this, a recent model study proposed that abnormal delta rhythms in DoC arise from altered neuronal firing patterns and reduced synaptic weights (Wang et al., 2025). Conversely, diminished alpha power reflects the characteristic global cortical suppression observed in severe diffuse postanoxic injury (Colombo et al., 2023).

Beyond the decreased delta power, we observed significant increase of center frequency and decrease of peak power from UWS to MCS. Moreover, both metrics showed significant correlations with the CRS-R score. Such correlations have also been reported for the peak frequency of the raw spectrum in a previous study. According to a visual categorization of EEG in DoC named “ABCD” model, the power spectrum could reflect the levels of structural or functional deafferentation that occur in patients with DoC (Timofeev et al., 2001; Frohlich et al., 2022). For instance, the UWS patients with dominant frequency <1 Hz (A type) could suggest complete deafferentation of thalamocortical connectivity. While the EEG power spectrum with a peak in the 5–9 Hz range or higher frequency ranges are associated with preserved thalamocortical projections. This form of shift can also be linked to the transitions from UWS to MCS or higher consciousness levels. Overall, the alterations of periodic spectrum also support the conclusion that the loss of consciousness are accompanied by the trend of EEG slowing (Lutkenhoff et al., 2020).

Despite historical neglect, the EEG spectral exponent is increasingly recognized as a marker of altered consciousness (Maschke et al., 2025). Beyond periodic activity differences between UWS and MCS, we observed significantly higher aperiodic exponents in UWS patients, reflecting steeper spectral decay—a distinct form of EEG slowing. As spectral exponents are theorized to index neural E/I balance (Gao et al., 2017; Wiest et al., 2023), and consciousness requires optimal E/I ratios (Gao et al., 2017; McKeon et al., 2024; Lord et al., 2023), severe brain injury may disrupt this balance (increased inhibition/reduced excitation). This shift could push the brain away from criticality, steepening spectral slopes. Additional biophysical factors—including active membrane currents and dendritic calcium spikes—may further shape EEG spectra by altering synaptic kinetics (Gao et al., 2020; Reimann et al., 2013; Suzuki and Larkum, 2017; Brake et al., 2024). Critically, exponent decreases from UWS to MCS occurred globally across all channels, suggesting whole-brain alterations in E/I balance or synaptic function. This effect was more pronounced in the 0.1–13 Hz range, indicating heightened sensitivity of low-frequency aperiodic activity to consciousness states. Such frequency-specific aperiodic-periodic interactions may explain why conventional power spectra often reveal changes in delta, theta, and alpha bands.

In addition to the comparison of spectral parameters averaged across channels or epochs, we also explored the spatiotemporal variabilities of these metrics. Our results showed significant increased spatial variability of delta power and spectral exponent with consciousness levels. This is in line with a previous study that reported reduced stability of brain hubs and heterogeneity of brain dynamics with the loss of consciousness (López-González et al., 2021). A possible explanation is that the local dynamics are strongly determined by the structural connections in low-states of consciousness, while local dynamics in conscious state can dissociate from their structural constrains and presented a diversity across the brain regions (Panda et al., 2022; Luppi et al., 2023). Moreover, positive correlation between the temporal variability of spectral exponent and consciousness levels or behavioral performance could be found in this study. This could be associated with the finding that the brains of unresponsive patients showed primarily less complex patterns and had smaller chances to transition between patterns (Demertzi et al., 2019; Cavanna et al., 2018; Cai et al., 2020; Bai et al., 2021a,b). Indeed, a previous study has reported the existence of spatial and temporal hierarchical differences of neural activity within the macaque cortex, which is modulated by the loss of consciousness (i.e., anesthesia). Such disruption of brain hierarchy corresponds to poor and rigid, structurally driven brain dynamics (Signorelli et al., 2021).

Furthermore, we investigated the diagnostic power of periodic and aperiodic features in detecting consciousness levels. Our results showed that both periodic and aperiodic metrics could effectively discriminate MCS from UWS with much better performance than that of a coin toss, while the aperiodic features yield better classification performance (higher accuracy and AUC) than the periodic features on the whole. This suggest that the aperiodic activities exhibit stronger modulation than oscillatory components under loss of consciousness and contains richer information about conscious states. Among all the aperiodic features, the temporal variability of spectral exponent demonstrates superior classification performance, strongly supporting its potential as a diagnostic biomarker for conscious states. Interestingly, the combination of periodic and aperiodic features could achieve better performance than those with only periodic or aperiodic features, especially in the detecting of MCS patients. This highlights the distinct physiological significance of periodic and aperiodic activity in DoC.

Several limitations of the current study should be acknowledged. First, although our sample size is comparable to those of previous EEG studies involving patients with DoC (Liu et al., 2023; Toppi et al., 2024), the relatively limited number of participants may affect the generalizability of the classification model. Future studies with larger, multi-center cohorts are required to validate our findings and build more robust and generalizable models for the diagnosis of patients with DoC.

Second, the present study separated the DOC patients into UWS and MCS, without accounting for more nuanced clinical and behavioral subtypes, such as MCS* patients (Thibaut et al., 2021), UWS patients who demonstrate covert auditory localization (Carrière et al., 2020; Aubinet et al., 2019), or individuals exhibiting cognitive-motor dissociation (Bodien et al., 2024). Such heterogeneity may introduce variability within groups and obscure more subtle electrophysiological differences between these functionally distinct subpopulations. Although the proposed periodic and aperiodic features show promise for discrimination between UWS and MCS, their ability to distinguish these finer-grained phenotypes remains unexplored. Future studies with larger, phenotypically refined cohorts are needed to evaluate whether these EEG biomarkers can support more precise subtyping and individualized prognosis.

Finally, relying solely on frequency-domain features provides limited insight into the mechanisms of consciousness and may also limit the accuracy and generalizability of classification models. To achieve a more comprehensive understanding of DoC and improve predictive performance, future studies should integrate advanced computational approaches—such as dynamic causal modeling or time-resolved network analysis (e.g., Panda et al., 2023), alongside multimodal integration with structural neuroimaging, to enhance explanatory and predictive power in DoC.

## 5 Conclusion

This study provides insight into the roles of periodic and aperiodic activities in characterizing DoC. Our results revealed that the EEG spectral changes with the altered consciousness levels are not only driven by the periodic oscillations but also the aperiodic activities, which may demonstrate performance in distinguishing between UWS and MCS. In addition to the temporal or spatial averaged metrics, the spatiotemporal variability of aperiodic activities also contains important information about the consciousness states. These findings may advance the mechanistic understanding of DoC and provide new insights for the detecting of residual consciousness.

## Data Availability

The raw data supporting the conclusions of this article will be made available by the authors, without undue reservation.
